# Spontaneous Recurrent Pneumoperitoneum due to Opioid-Induced Constipation: A Case Report

**DOI:** 10.7759/cureus.8205

**Published:** 2020-05-20

**Authors:** Varun Kaushal, Behzad Amoozgar, Pavan Garala, Karim Bayanzay, Shuvendu Sen

**Affiliations:** 1 Internal Medicine, Jersey Shore University Medical Center (Perth Amboy), Perth Amboy, USA; 2 Internal Medicine, Jersey Shore University Medical Center (Perth Amboy), Perth Amboy, USA

**Keywords:** methadone, opiates, pneumoperitoneum, fecal disimpaction, stercoral colitis

## Abstract

Long-term treatment with or addiction to methadone and other opiates can lead to serious complications such as opioid-induced constipation (OIC). Here we report a case where a long-term opioid user presents in the ER in respiratory distress. Radiographic findings concerning pneumoperitoneum and cooperation with specialists lead to a diagnosis of stercoral colitis with possible micro-perforations. Through fecal disimpaction and counseling on chronic opioid use, the patient initially improved, but consecutively had a fatal outcome.

## Introduction

Opioid-induced bowel dysfunction (OIBD) is a debilitating adverse effect of chronic opioid use. Opioid-induced constipation (OIC) is the most common manifestation of OIBD [1]. Heroin, as one of the most prominent abused opioids, is not excluded from this adverse phenomenon. Regardless of the root of administration/abuse, chronic heroin users experience severe constipation [2]. Methadone, major substitution therapy for the treatment of heroin addiction is not excluded from this side-effect [3]. Severe constipation may lead to stercoral colitis, localized ischaemic necrosis of the colonic wall, stercoral ulceration, and eventual perforation [4-6]. However, the rate of colonic perforation significantly decreased due to radiologic workup and hence early diagnosis. Here we demonstrate a rare case of stercoral colitis, which presents with respiratory distress and pneumoperitoneum but without any colonic perforation [4-6].

## Case presentation

This is a case of a 31-year-old male with a past medical history of heroin abuse on methadone who presented to the ED with severe abdominal pain and shortness of breath. He stated that his last voluntary bowel movement was eight weeks ago and he had tried laxatives intermittently with no improvement.

**Figure 1:**
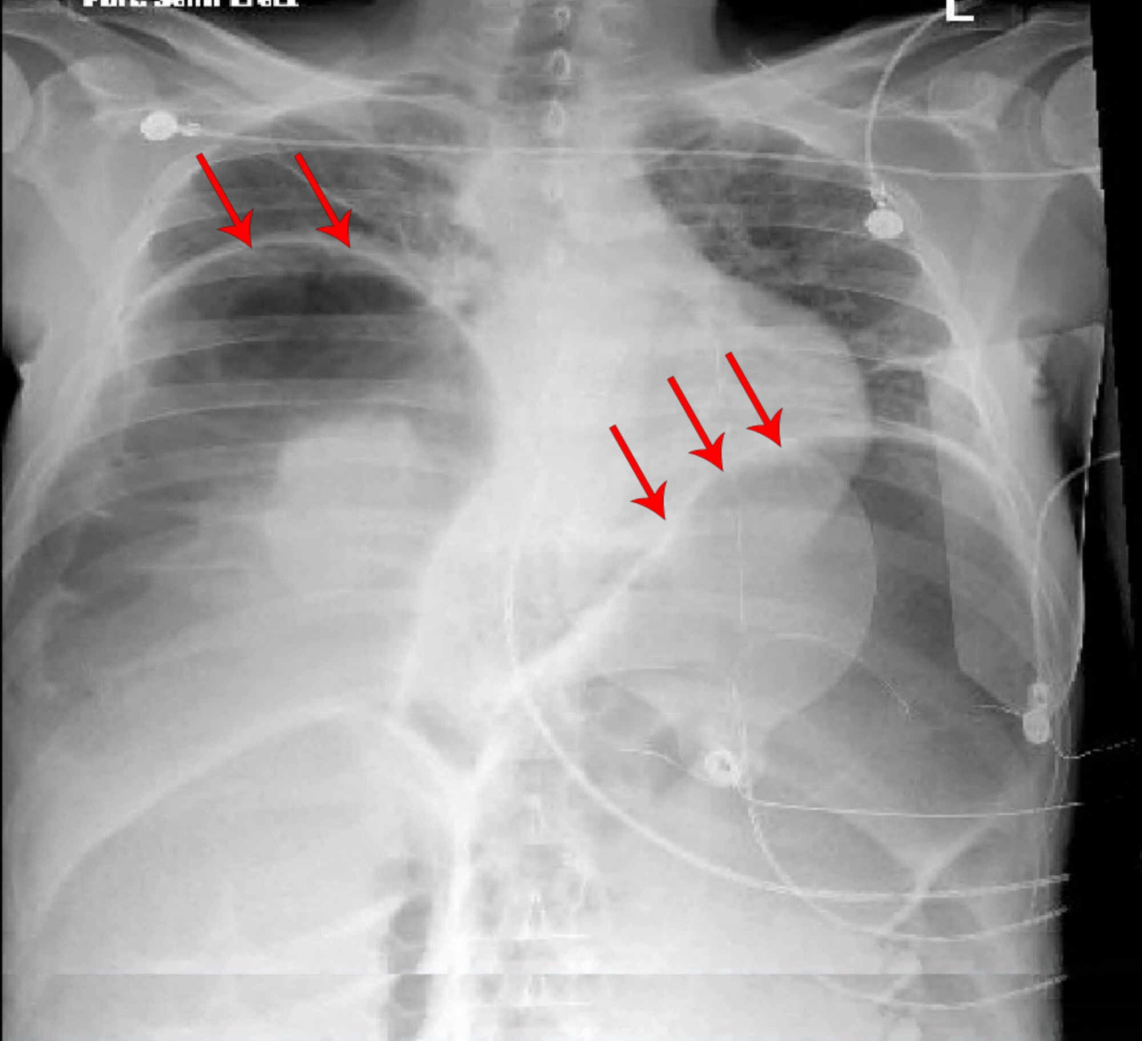
Portable chest X-ray depicting massive free air/tension pneumoperitoneum (red arrows on both sides).

**Figure 2:**
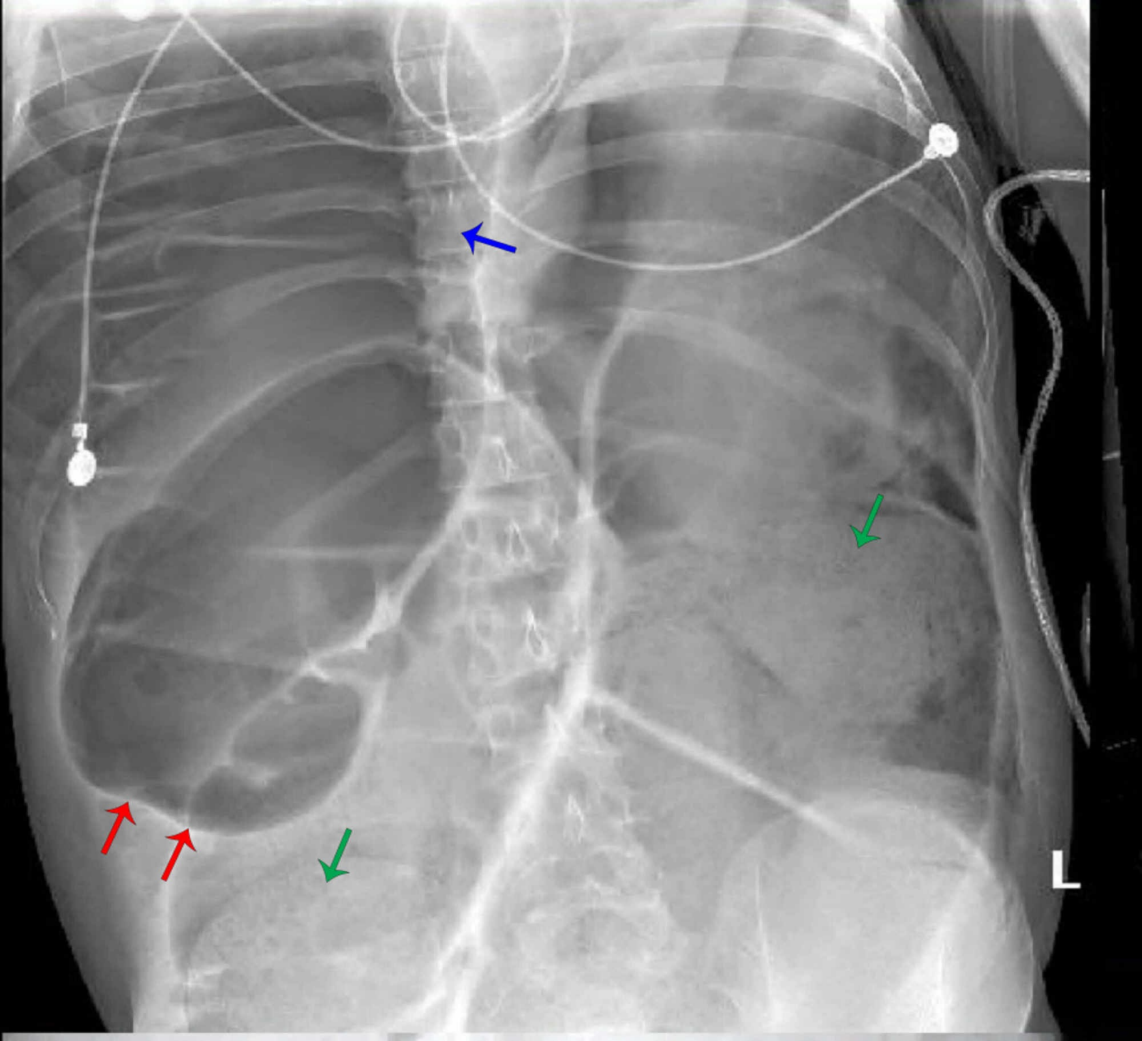
Abdominal X-ray showing diffuse marked distension of colon (red arrows) with pneumoperitoneum (blue arrrow), and large amount of stool (green arrows).

Two days before the current encounter, the patient had been experiencing fecal and urine incontinence requiring the use of a diaper. He stated that he had been inhaling heroin and 50 mg of methadone daily. He denied any nausea, vomiting, or diarrhea. The patient was previously admitted for a similar episode 10 months before for constipation which was relieved with disimpaction. During this admission, CT scan of the abdomen and pelvis with contrast was done which showed stercoral colitis, but no signs of perforation or peritonitis. Vital signs: blood pressure 122/87 mmHg, pulse 93 beats/min and regular, respiratory rate 18 breaths/min, temperature 98°F (36.7°C), and SpO2 99%. Laboratory workup showed white blood cell of 8.8*103/mL [4-11 10-3/mL], hemoglobin of 16 g/dL [13-17g/dL], potassium of 4.4 mmol/L [3-5.5 mmol/L], magnesium of 1.3 mmol/L [1.5-2.5 mmol/L], aspartate aminotransferase (AST) of 25 u/L [5-40 u/L], and alanine transaminase (ALT) of 12 u/L [5-40 u/L]. Urinalysis showed the presence of nitrites and trace leukocytes. During the physical examination, the patient exhibited abdominal distension, firm and generalized tenderness. The initial chest X-ray was suggestive of pneumoperitoneum (Figure 1). As a follow up, X-ray abdomen obstruction series was done which showed pneumoperitoneum with a large amount of free air beneath the right hemidiaphragm. Additionally, X-ray showed a massive amount of gas and stool throughout the colon (Figure 2). Based off the abdominal distention with tenderness along with imaging the diagnosis of intestinal perforation was made. Due to possible intestinal perforation and the presence of leukocytes on urinalysis (colon as the possible source due to history of fecal impaction), the patient was placed on piperacillin and tazobactam 3.375 g. Surgery and infectious disease services were consulted and the patient was taken to the operating room (OR) for exploratory laparotomy and repair of presumed bowel perforation. In the OR no perforation was seen, but instead, a distended colon and rectum were appreciated for which a manual disimpaction was done. The patient's condition improved after disimpaction and antibiotic therapy. The patient was discharged with amoxicillin-clavulanate 875/125 mg and to follow up outpatient.

Eight weeks later, he presented again to the ER with severe abdominal pain but with concomitant shortness of breath. His last bowel movement was three weeks prior to this encounter. Since then, he had been having gradual abdominal distension leading to abdominal pain and progressive respiratory distress. X-ray abdomen demonstrated massive free air/tension pneumoperitoneum. CT scan of the abdomen and pelvis with contrast showed marked dilatation of the colon, which was filled with a large amount of stool, resulting in a marked elevation of the right hemidiaphragm, similar to prior study. Moreover, a circumferential wall thickening of the rectum with impaction of stool within the rectosigmoid colon was detected which were suggestive of stercoral colitis.

Surgery and gastroenterology consultants were called and they recommended enema and stool disimpaction. Fleet and soap suds enemas were administered but with no improvement. He was taken to the Endoscopy Unit for flexible sigmoidoscopy disimpaction under light sedation. While in the endoscopy room concomitant colonoscopy was done which showed dilated colon with erythematous mucosa, but no ulcerations were noted. Furthermore, detox service was consulted, and the dose of methadone was decreased and the patient was advised to ultimately discontinue his opioid use. The patient's condition improved and he was discharged with docusate sodium and followed up with a detox program. The following year the patient again presented with similar symptoms and on arrival to the ER the patient had cardiac arrest and passed away.

## Discussion

The common opioids adverse effect on the gastrointestinal system is increased segmental motility and decreased peristalsis resulting in a phenomenon called OIC [7-8].

OIC is a common problem in patients on chronic opioid therapy and mainly manifests as constipation, but also may be accompanied by nausea, bloating, early satiety, and pain. Approved and safe treatments for OIC include methylnaltrexone, naloxone, naldemedine, and lubiprostone [7-8].

One of the most prominent side effects of chronic heroin abuse is severe constipation. Methadone is a long-acting opioid agonist that binds to μ-opioid receptors with higher intrinsic activity than morphine, but lower affinity [9-10]. In substitution therapy for the treatment of heroin addiction, methadone is the first choice. Similar to heroin, chronic methadone therapy may result in severe constipation or OIC [9-10].

Fecaloma is a mass of hardened and impacted feces which mostly accumulates in sigmoid colon and the rectum as a result of chronic severe constipation or OIC. Hard fecaloma causes distension and increased pressure on the colon wall and decreases vascular perfusion at the antimesenteric side. The presence of fecal impaction material in colonic lumen associated with inflammation and distension of the affected colon segment is called stercoral colitis [11-12].

Inflammation and increased intraluminal pressure eventually may lead to ulceration and perforation. Evident of obvious or silent perforation may result in pneumoperitoneum. Interestingly, in our case, no perforation was detected both by surgical intervention and colonoscopy. We postulate that stercoral colitis may result in micro-perforation and accumulation of gas and hence manifests as pneumoperitoneum. It is crucial to diagnose and address stercoral colitis and prevent bowel perforation [13-16].

Besides perforation, severe constipation may indirectly exhibit respiratory symptoms. This mainly includes, but not limited to, opioid-induced respiratory depression [17]. Moreover, few publications have described respiratory distress as a result of severe constipation; upward pressure imposed on the diaphragm by constipated and distended bowel may result in increased anteroposterior pulmonary diameter, decreased lung height, and a reversible restrictive pulmonary disorder similar to our case [18-19]. Hence, it is pertinent to consider the aforementioned unusual respiratory presentation, specifically while severe concurrent constipation exists. 

## Conclusions

We describe the unusual presentation of pneumoperitoneum without evidence of an obvious perforation or ischemia noted on colonoscopy. Providers must be aware of the risk of recurrent pneumoperitoneum due to ongoing opioid or methadone use and which may result in severe adverse or fatal outcomes. Severe constipation caused by chronic opioid use or abuse may lead to perilous adverse events such as bowel perforation. One should consider possible perforation if signs such as stercoral colitis and pneumoperitoneum are detected in imaging studies. Apart from abdominal signs, opioid usage may result in respiratory side effects. Paradoxical respiratory distress may manifest as the result of increased pressure induced by constipated bowel. These clinical scenarios should warn clinicians about the severity of the presentation.
